# Game-Based Promotion of Assertiveness to Mitigate the Effects of Bullying in High School Students: Development and Evaluation Study

**DOI:** 10.2196/58452

**Published:** 2024-12-24

**Authors:** Francisco Lepe-Salazar, Fernando Mejía-Romero, Dámaso Benicio-Rodríguez, Aylin Hernández-Reyes, Tatsuo Nakajima, Sarita Salgado-Torres

**Affiliations:** 1 Ludolab Colima Mexico; 2 Universidad de Colima Colima Mexico; 3 Department of Computer Science and Engineering Waseda University Tokyo Japan

**Keywords:** serious games, bullying, assertiveness, multiple composite scenarios, scenario, cognitive behavioral therapy, gaming, design, development, bully, assertive, feasibility

## Abstract

**Background:**

For years, Mexico has reported the highest global incidence of school bullying, with approximately 19% of students going through some form of hostile peer interactions. Despite numerous interventions, these harmful conducts remain deeply entrenched in educational environments.

**Objective:**

To address this issue, we developed *Bernstein*, a serious game that promotes assertiveness—an essential protective factor that reduces the negative effects of bullying.

**Methods:**

*Bernstein* was designed using multiple composite scenarios, a methodology grounded in cognitive behavioral therapy. To evaluate the game’s effectiveness, we conducted an exploratory trial using the Rathus Assertiveness Schedule to assess assertiveness levels before and after the intervention. Participants were high school students who met the inclusion criteria of being open to playing a serious game (with no prior gaming experience required) and having access to a computer with internet connectivity for remote participation. A total of 100 students (65 boys and 35 girls) enrolled in the intervention; however, only 46 participants in the treatment group and 46 in the control group completed the study, resulting in a dropout rate of 8% (8/100). A paired 1-tailed *t* test was used to compare pre- and postintervention scores within each group, and a one-way ANOVA was conducted to compare the average score improvements between the 2 groups.

**Results:**

The treatment group showed a pretest mean Rathus Assertiveness Schedule score of –2.78 (SD 25.93) and a posttest mean of 1.69 (SD 29.48), with a paired 1-tailed *t* test yielding a *P* value of .01. The control group had a pretest mean of 2.07 (SD 25.69) and a posttest mean of –2.39 (SD 32.98), with a paired 1-tailed *t* test yielding a *P* value of .04. The one-way ANOVA (between groups) yielded a *P* value of .006, indicating a statistically significant difference, favoring *Bernstein* over the alternative protocol. Participant feedback highlighted the game’s engaging narrative and character design, although usability issues, such as navigation challenges, were noted as areas for improvement.

**Conclusions:**

The results suggest that *Bernstein* is a promising tool for promoting assertiveness in high school students, providing a potential strategy for addressing bullying-related issues. The study underscores the value of integrating *Bernstein* into educational programs, offering students a safe and interactive environment to develop resilience. As an exploratory trial, this study faced limitations affecting the generalizability of findings, including the remote format’s impact on facilitator guidance and a relatively small sample size. Further trials with larger, more diverse groups are recommended to validate these early results and enhance *Bernstein*’s scalability as part of a comprehensive antibullying strategy.

## Introduction

### Background

“Bullying” is a term used to describe the aggressive and intentional acts carried out by an individual or a group repeatedly against an individual [1]. Ever since bullying was recognized as a psychosocial problem, several research groups have investigated its causes, as well as possible ways to eradicate it [2,3].

It commonly takes place in spaces where people spend time together (eg, school, work, etc). With the advancement of technology, new forms of bullying have emerged, such is the case of cyberbullying [4]. An example of cyberbullying is online harassment. In this form of digital bullying, the abuser has the advantage of being able to invade the personal space of the person being bullied at a distance, keeping his or her real identity anonymous, which in turn allows him or her to provoke greater fear in the person being bullied [5].

Those who have experienced public humiliation, including bullying and cyberbullying, are considered to be more likely to commit suicide [2]. Therefore, worldwide, multiple programs and initiatives have been carried out to counteract its effects. However, as studies show, this problem continues to grow [6].

According to information presented by the Organization for Economic Cooperation and Development, Mexico has held the first place in cases of bullying at school on various occasions [7,8]. In 2019, the results of the Programme for International Student Assessment test indicated that approximately 19% of students in the country had experienced some form of bullying. Furthermore, it was stipulated that a significant number of suicides committed by children in Mexico were due to cases of bullying, and, unfortunately, these numbers are expected to increase [9].

To contribute to the solution of this issue, we started a new project. A recent analysis of interventions to prevent bullying in schools showed that enhancing interpersonal skills, particularly assertiveness, stands out as the most effective protective factor in mitigating its consequences [10]. With this insight, we decided to develop a solution that promotes said protective factor.

To appeal to a young demographic, we chose to work with serious games [11]. A serious game differentiates from regular games in that they have an additional objective that can be educational or illustrative [12].

To ensure that the protective factor is properly addressed, we made use of the multiple composite scenarios (MCS), a methodology for the design of serious games rooted in principles of cognitive behavioral therapy (CBT) [13]. Unlike other game design approaches, MCS offers a unique blend of versatility and specificity, providing a comprehensive framework for crafting immersive experiences that properly address mental problems. The game we created with it is called *Bernstein*.

*Bernstein* is a serious game that promotes the development of assertiveness in high school students. To assess its potential benefits, we carried out an exploratory trial. This paper outlines the findings from this intervention, delving into its benefits and limitations. Furthermore, we discuss our plans for future work aimed at enhancing its overall effectiveness.

### Notions of Bullying

Bullying is the deliberate, intentional, and repeated use of force, threats, and coercion to abuse; cause fear or distress; dominate; harm; exclude; and intimidate physically (eg, slapping, hitting, and kicking), verbally (eg, threatening, yelling, and insulting), and psychologically (eg, denigrating and belittling) an individual or a group of people who are unable to defend themselves [14-19]. Bullies (ie, perpetrators of the aggression) usually choose their targets based on characteristics such as appearance, disability, age, race, religion, sex, and so forth [18,20].

People become bullies for various reasons including imitation, having a low self-esteem, or experiencing violence at home [21-23]. The person most affected by bullying is the one who receives the aggressive acts, that is, the recipient. As a consequence of bullying, those who are bullied may develop depression, drop out of school, or commit suicide [2,17,24].

Different actors intervene in bullying in addition to the perpetrator and the recipient, including facilitators (those who join the bully to antagonize others), reinforcers (those who do not antagonize but indirectly support the bullying by either making fun of the target or encouraging the bully), bully+target (those who are both a bully and a recipient of bullying), defenders (those who in some way help the target), and bystanders (those who witness the bullying but do not act accordingly) [25]. It should be noted that these extra actors are not necessarily present on every occasion.

Given its prevalence in modern societies (from 17% to 20.8% in some sectors), bullying is considered a sociocultural phenomenon [15,18]. It is believed that more than 50% of students worldwide have been involved in some form of bullying, as targets, bullies, or bystanders [17].

Some of the strategies that exist to deal with this phenomenon include school programs, institutional policies, rules within the classroom, teacher training, information campaigns, awareness campaigns, discussion forums, workshops, psychological interventions, and educational interventions, among others [15,17].

The type of consequences experienced by the recipients of bullying depends on the severity of the attacks. There are cases in which the recipients do not experience consequences in the mid- to long term. This is due to a series of factors that are directly related to the level of affectation of the recipient. That is, the greater the presence of certain factors, the stronger or weaker the consequences may be. These types of factors are known as risk factors (those that reinforce the negative effects) and protective factors (those that reduce the negative effects).

### Selection of the Protective Factor to be Promoted

To learn which factors related to bullying cause the greatest affectation in young students, we conducted a review of the literature. Furthermore, we searched for factors that may help reduce the affectation by this psychosocial phenomenon (ie, protective factors). For this task, we consulted various mental health articles related to interventions carried out in schools [1,2,21-23,25,26].

We found that having a low self-esteem and social isolation are 2 of the most relevant risk factors. In contrast, having a greater number of social relationships, being assertive, and having a high self-esteem were shown to be the most prominent protective factors in reducing the negative effects of bullying. It should be noted that we found that increasing self-esteem is effective in elementary school children but not as effective at older ages. Given that our target audience are high school students, for our project, we chose to focus on the promotion of the protective factor of assertiveness.

Assertiveness should not be confused with aggression, which is the forceful or disrespectful expression of one’s way of seeing things, or the imposition of one’s needs onto others. Assertiveness is an essential, interpersonal behavior that aims to enable individuals to (1) minimize power differences that may exist with others; (2) act in their own best interests; (3) stand up for themselves without undue anxiety; (4) express their beliefs, concerns, feelings, interests, needs, opinions, point of view of things, and personal rights honestly; (5) exercise their rights; and (6) share privately held information with people in a position of authority [27-29]. This while respecting and acknowledging the rights of others.

### Games and Bullying

Ours is not the first project that aims to address a psychosocial issue such as bullying by making use of a video game. Video games offer players the opportunity to try different paths and learn different points of view and perspectives in digital environments [30]. For that reason, it is believed that they are capable of promoting behavior change, problem-solving, and acquisition of skills.

The main goal of commercial video games is to entertain. Games whose purpose goes beyond entertainment are referred to as serious games. Serious games differ from commercial games in that they have an additional objective [12]. Among the objectives that these usually pursue are raising awareness, teaching, training, changing perceptions, persuading, promoting skill development, improving attitudes, instilling positive attitudes, nurturing player empathy, and so forth [16,18,30-41].

To achieve this goal, serious games make use of formal strategies and techniques. Such is the case of serious games for education, which make use of pedagogical methodologies to teach a concept or skill [42]. Other topics covered by serious games include caring for the environment, mental health, and sports, among others [11,13,43,44].

There are various game titles related to the subject of bullying. StopBully is a serious game that through role-playing puts the player in the role of recipient or bystander, thus simulating real-world situations [16]. Its objective is to prevent bullying by helping recipients improve their behavioral competence to avoid or face situations [39].

Conectado is an educational game that seeks to increase awareness among young people about school bullying [31,32]. This title puts players in the role of recipients with the aim of fostering in them empathy with those who are bullied.

FearNot! is a serious game that takes place in a virtual school with characters typical of everyday school life (eg, bullies, recipients, bystanders, and helpers) [35]. Its goal is for players to observe bullying scenarios and interact with the recipients, as well as advise them on the best way to deal with the situation.

Stop bullying now! is a serious game whose goal is to enable caregivers of people with disabilities to learn how to respond to bullying situations [18]. To do this, it presents different characters and situations, such as children and adults with disabilities, parents, teachers, supervisors, and bullies who show different bullying behaviors.

Happy Class is a role-playing game (RPG) in which students experience the role of bullying bystanders through chat [41]. Its goal is for players to understand the seriousness of the damage caused by cyberbullying and the important role of bystanders while learning how to prevent further violence by taking appropriate action.

The game studio Disparity developed and published an indie title that covers the topic of bullying called “Ninja Pizza Girl” (NPG). NPG narrates the story of a 16-year-old girl named Gemma, who works in her father’s business delivering pizzas and who is forced to face “the most ruthless enemies a teenage girl can have: other teenagers” [45]. Throughout the story told in NPG, topics such as self-esteem, bullying, and resilience are discussed. Enemies in this game attack the player through insults and taunts. An interesting detail about the production of NPG is that part of the team that worked on the game had previously been a recipient of bullying [46].

A Day at my School is a serious game developed with Unity that highlights the perspective of a bystander and that presents a situation in which the abuser becomes the recipient. This title consists of 2 stages whose objective is to raise awareness and motivate the player to report bullying when the situation arises [47]. It should be noted that this serious game is available for free on the web.

A research group at Leuphana University presented the conceptual design for Bully you, Bully me, a serious game that asks players to enact scenarios of bullying to make them reflect about the consequences of their actions [48].

There are also commercial games that deal with bullying. Such is the case of Bully, an action and adventure game set in an open world environment [49]. In Bully, the player plays a 15-year-old teenager who can decide whether or not to harass others and see what effects and consequences his actions have. It should be noted that its effectiveness in preventing bullying has not been tested.

In general, serious games that address bullying tend to (1) simulate real-world scenarios; (2) present cases of bullying; (3) provide recommendations; (4) ask the player to take action (eg, supporting the recipient of bullying); and (5) optionally, encourage reflection. Furthermore, several of the titles presented here contemplate their use as part of an intervention in which a teacher or a guide uses them as an example to invite participants to dialogue and reflect on it.

Among the limitations of the aforementioned examples are (1) little variety of scenarios or narratives, (2) little variety of devices on which they are available, (3) little variety of actions that they allow players, and (4) type of audience to which they are directed (mostly children and young people).

Inspired by other games that seek to contribute to the mitigation of bullying, we decided to present realistic scenarios that allow players to act and reflect on it. However, unlike them, we decided to (1) create a game that would present different scenarios (eg, bullying between people in the same family and professional bullying), (2) work on a prototype that can be used on different devices, (3) increase the number of actions a player can perform, and (4) create a game that is interesting for young and older players. Another key distinction is that, to the best of our knowledge, our serious game is the only one explicitly designed to promote assertiveness in players as a strategy to mitigate the impact of bullying they may experience. In other words, *Bernstein* uniquely frames assertiveness as a practical tool for building resilience against bullying, rather than treating it as a stand-alone skill or ultimate objective.

### Notions of Assertiveness

Assertiveness refers to a person’s ability to (1) communicate with others in a clear, firm, positive, honest, direct, and balanced way; (2) stand up for his or her own beliefs without limiting or attacking others; (3) meet his or her own needs and concerns; (4) demonstrate certainty in his or her actions; (5) express what he or she feels and thinks, even if it results in negative consequences; (6) be direct in asking for what he or she wants or needs; (7) behave confidently; (8) be sociable and active; (9) take control of his or her life; and (10) being frank [50-57]. This differs from aggressiveness in that an assertive person does not seek to impose himself or herself or harm others to achieve his or her goals. Assertiveness is commonly linked to job success, as well as personal growth and development [54,55].

### Relevance of Assertiveness in Adolescence

Adolescence is a pivotal stage for socioemotional development, during which young people navigate complex identity formation and peer dynamics. In this process, the ability to express thoughts, needs, and emotions clearly—especially when faced with peer pressure or conflict—becomes essential. Assertiveness equips adolescents with the confidence and resilience to advocate for themselves while fostering positive relationships [27,58]. As a key protective factor, it may not only help reduce the risk of bullying but also enhance academic performance and strengthen social connections.

### Games and Assertiveness

There is a wide variety of game-based approaches that seek to contribute to the development of assertiveness in players. Such is the case of the model presented in the study by Cheong et al [52] for conflict resolution, which is based on balancing the assertiveness and cooperation of users. In the study by Hendrix et al [59], a proposal is presented to use games to increase children’s social competence, encouraging them to talk about their needs. In contrast, in the study by Eng [60], a learning strategy based on games is disclosed to teach assertive communication and improve team play. van der Lubbe et al [61] created a serious game to train the verbal resilience of the players, in order to avoid scams. Finally, in another study by van der Lubbe et al [62], a proposal for the use of serious games is presented to empower vulnerable groups by promoting the development of skills such as assertive communication.

In general, these approaches are characterized by presenting activities that (1) promote the development of an interpersonal skill (eg, assertive communication) and (2) allow players to practice said skill through game scenarios. Unlike these examples, our approach goes beyond fostering a single skill; it aims to encourage holistic improvements in players’ mental health by enhancing their cognitive and behavioral patterns.

## Methods

### Implementing MCS

#### Overview

We set out to develop a serious game aimed at promoting assertiveness in students. To achieve this, we used the MCS methodology, which enables the design of games tailored to prevent specific mental disorders [13]. This adaptability (ie, the ability to customize tasks, scenarios, dialogues, feedback, etc) is crucial, as the efficacy of a game depends on its capacity to address the specific needs and challenges of its serious goal.

MCS draws on CBT principles to reduce the impact of risk factors and promote the adoption of protective factors [13]. CBT is a psychological treatment that helps reframe negative emotions, thoughts, memories, and ideas [63,64]. Both MCS and CBT rely on techniques such as Socratic questioning, definition of terms, cost-benefit analysis, and systematic gradual exposure to achieve their objectives.

Various technological applications (eg, computer software, mobile apps, wearable sensors, and virtual agents) have been inspired by certain aspects of CBT. Such is the case of (1) technology for user data collection, which is used to collect and analyze data such as mood changes or facial expressions; (2) self-help software, which enables patients to address mental health problems by presenting them psychoeducational materials and activities; (3) technology-based activities, which aim to facilitate activities under therapist guidance; (4) psychoeducation online platforms, which deliver psychoeducational content through multimedia to enhance patient understanding of their disorder; and (5) technology for awareness and coping, which aims to raise awareness about mental health and help individuals deal with life’s challenges through specific tasks [13].

It is important to note that the MCS methodology differs from these applications by focusing on preventing mental disorders through health games. MCS integrates the complete CBT protocol within the game itself, ensuring that players engage in meaningful experiences aligned with therapeutic goals.

#### Risk Versus Protective Factors

A risk factor is a determinant or associated variable that increases the chances that a person will be affected by a problem. In contrast, a protective factor is a determinant or associated variable that reduces the chances that a person will be affected by a problem. For our project, the risk factor we used was social isolation, and the protective factor we promoted was assertiveness.

#### Promoting a Protective Factor With a Serious Game

To promote the adoption of a protective factor, MCS makes use of game scenarios that illustrate the recovery process of a virtual patient. The steps of MCS are to (1) *introduce* a risk factor to the player, (2) *depict* the effects that the risk factor has on a virtual patient, (3) *inform* the player about important psychoeducational facts through trustworthy characters, (4) *distract* the player with game dynamics, (5) *evaluate* what has been learned with game activities, (6) *defend* the virtual patient, (7) *reflect* on the benefits of the protective factor, (8) *apply* the knowledge learned in the virtual world, and (9) *involve* the player in the recovery of the virtual patient [13].

Through the creation of varied and dynamic scenarios, players are presented with a rich and immersive setup. This not only captures their attention but also ensures the adoption of positive behaviors.

To illustrate the consequences of risk factors in a nonthreatening manner, the figure of the virtual patient is leveraged. This approach is designed to avoid making players feel criticized or judged. In our game, we decided to have secondary characters fulfill this role. Our intention was to provide players with an opportunity to interact with various nonassertive characters, enabling them to witness firsthand the consequences of such behaviors.

Another strategic element contributing to the overarching goal is the unique scoring system it proposes: Love Coins. Love Coins operate as follows: players who actively engage in positive behaviors earn higher scores. This system was designed as a way to encourage players to adopt healthy behaviors both within the game context and, possibly, in the real world.

### Designing Our Game

#### Overview

To align with different player interactions, scenarios were designed to gradually increase in complexity. They ranged from practicing simple self-expression to managing more challenging interpersonal conflicts. Some scenarios required players to engage in assertive dialogue with in-game characters, while others involved problem-solving tasks that fostered collaboration and interpersonal growth.

Each scenario was crafted to mirror real-life social dynamics, reflecting challenges that players might encounter outside the game. Characters’ responses were programed to adapt based on the player’s choices (eg, rewarding assertive behavior and gently redirecting passive or aggressive actions). This dynamic feedback system, integrated with the Love Coins scoring mechanism, gave players the opportunity to experiment, reflect, and adjust their behavior in real time.

Furthermore, gameplay elements such as timed responses and branching dialogues ensured that the learning process remained engaging while providing measurable feedback on behavioral changes throughout the gameplay.

#### Bernstein

**Figure 1:**
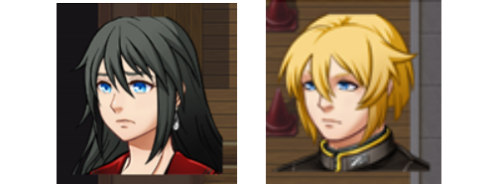
Avatars for the main character.

**Figure 2:**
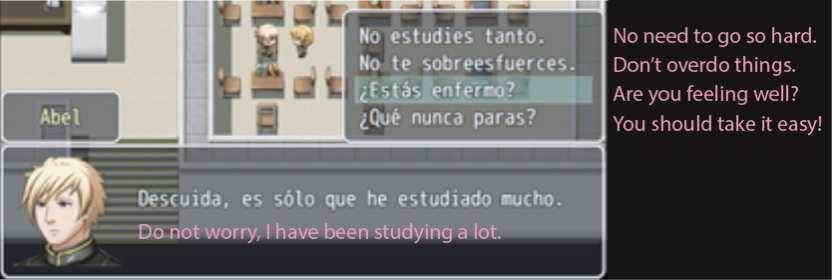
Example of a conversation.

**Figure 3:**
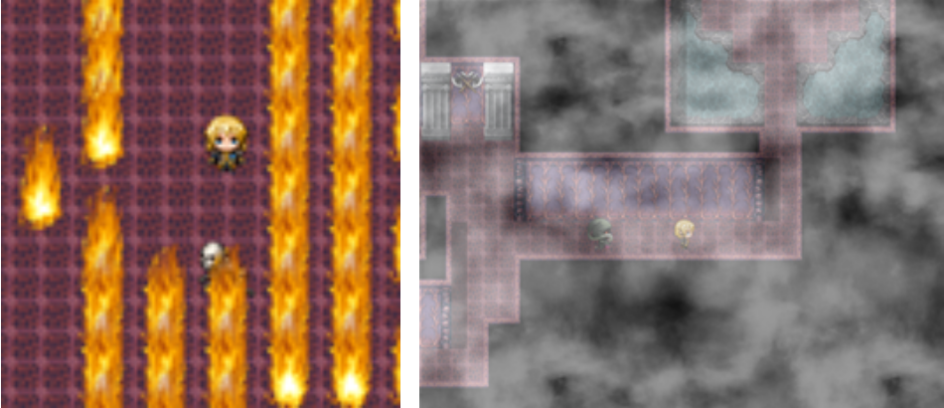
Example of the puzzles: Danger Room (left) and Confront Monster (right).

The name of the title we created using MCS is *Bernstein*. *Bernstein* is an RPG with features such as puzzles and exploration (Figures 1-3). The plot of our serious game focuses on rescuing a friend who is trapped in a dungeon (Figure 3). To do so, the player must enlist the help of people who are very socially isolated and not assertive. *Bernstein*’s prototype was created with the tool RPG Maker.

#### Narrative

The plot of the game takes place at the Bernstein Institute, a boarding school located in what was previously a castle. Rumors recently spread among the students, speaking of the existence of a hidden treasure somewhere in the underground dungeon, a restricted access section of the facilities. This draws the attention of Leopold Stotch, the protagonist’s classmate, who decides to venture out on a treasure hunt.

What Leopold did not know before embarking on a quest is that this dungeon was designed with various mechanisms to ward off intruders, some of supernatural origin. For that reason, he gets trapped with no apparent way out. The game begins the moment the player receives a call for help from Leopold. To get to where his or her friend is, the player must make his or her way through obstructed paths. To do so, the player will need specific tools. Fortunately, everything needed by the player can be found within the institute itself. However, the player will have to convince the owners of said tools to cooperate.

**Figure 4:**
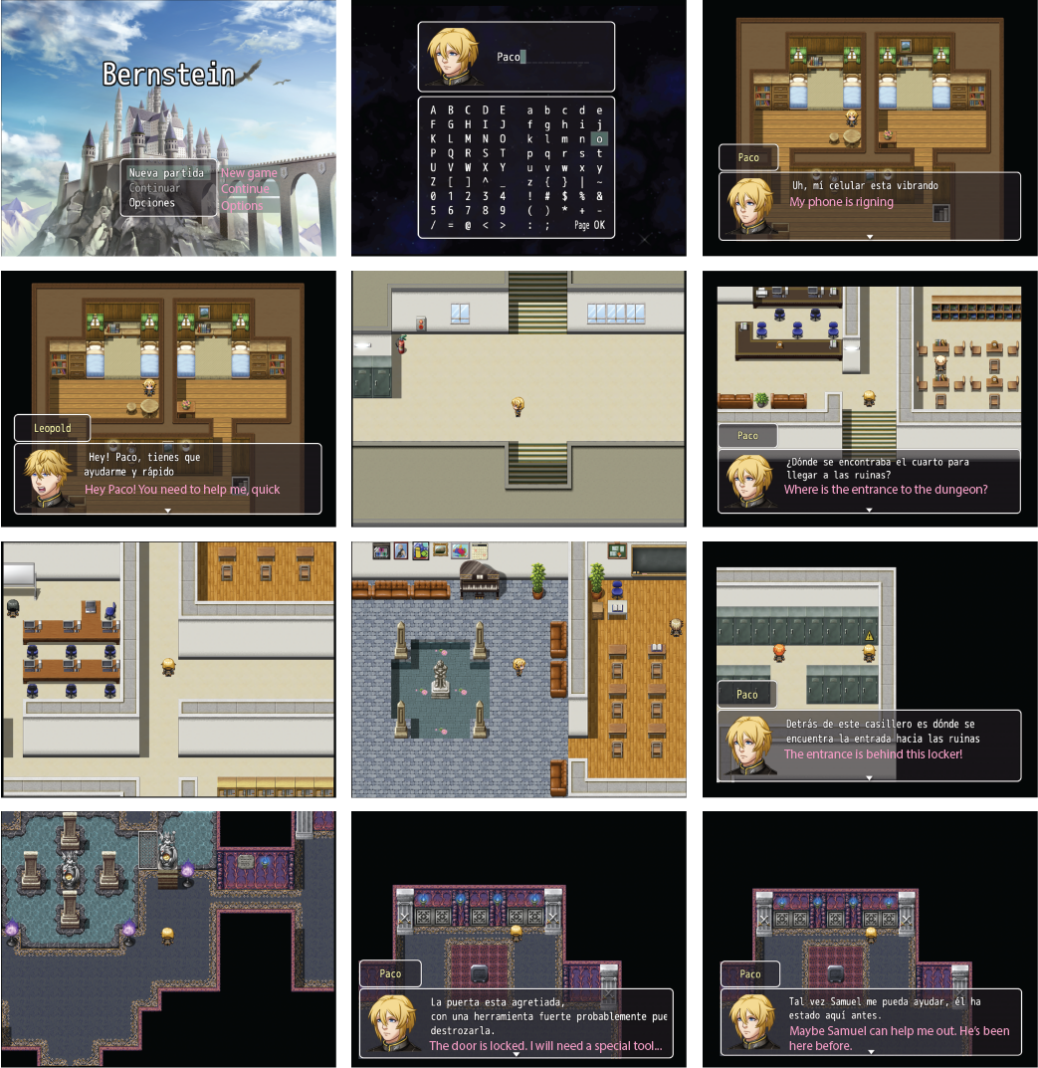
Walkthrough of the game.

To win, the player must help his or her new friends to overcome their insecurities and become more assertive. By doing so, the player can get the items needed to enter the section of the dungeon where Leopold is trapped. A walkthrough of the game is shown in Figure 4.

#### Gameplay

The game’s map is divided into 2 sections: the boarding school and the dungeon (Figure 4). The boarding school is an open area, which the player can freely explore. It is in this section where the classmates who can help the player in his or her adventure are found. In contrast, the dungeon is a closed space. It has 6 doors that can be opened with a different object. Five of these doors, when opened, lead to a puzzle that the player must solve. The sixth door is where Leopold is trapped, so opening it ends the game.

To win, the player must (1) earn many Love Coins by helping his or her new friends to be more assertive, (2) get specific items to open doors, and (3) solve the puzzles. The game has 3 different endings (good, regular, and bad). Which one the player gets depends on the score he or she obtains.

#### Game Scenarios

To facilitate the understanding of the MCS present in the game, we summarized the content of the game in Table 1. The first rows present the name of the characters and the object they possess. The subsequent rows break down the steps of MCS for each character.

**Table 1:** Game scenarios present in Bernstein.

**Character**										
			Felix	Abel		Samuel		Elena		Beatriz
**Specific object**										
			Wrench	Book of glyphs		Pickaxe		Battery		Stethoscope
**Step**										
	Step 1: Present risk factor	Player learns that the character believes that things will get awkward or something will go bad if he tells others what he wants/needs		Player learns that the character tries to be better because he feels obliged to	Player learns that the character thinks that what he likes is not as important and no one cares		Player learns that the character is a perfectionist, she wants to do everything the “right” way		Player learns that the character sees everything in a chaotic or catastrophic way	
	Step 2: Show consequences	Character has no friends, avoids talking to others		Character demands a lot from others and is unhappy	Character is very pessimistic		Character sees flaws as unacceptable, even her own		Character is constantly stressed about everything	
	Step 3: Psychoeducate	Discussion topic: We cannot know everything with absolute certainty, we have to be open to try new things		Discussion topic: One should do things that one enjoys and likes	Discussion topic: Learn to see the good things about oneself		Discussion topic: Things can be good without being perfect		Discussion topic: Learn to tolerate uncertainty	
	Step 4: Distract with activities	Talk about machines		Talk about hobbies	Talk about treasures in the boarding school		Talk about her camping trips		Talk about his piano lessons	
	Step 5: Evaluate what was learned	The player must try to check whether what the character thinks is true		The player must highlight the importance of not pleasing everybody	The player must mention his or her successes		The player must identify the negative and positive side of things		The player must analyze the situation realistically	
	Step 6: Defend the virtual patient	A character appears saying that it will not go well for him		The character’s older brother appears to remind him of his duties	A colleague points out that the world is cruel and goals are not easily met		The character begins to worry when she sees her leaves blown by the wind		The character receives a phone call about a new project	
	Step 7: Reflect on benefits	Interacting with others may be better than you expect		You would feel better saying “I would like to...” instead of “I have to...”	Your qualities do matter		Rarely is something all good or all bad		Sometimes things look bad because you feel bad about them	
	Step 8: Apply knowledge	Puzzle: Danger Room		Puzzle: Confront Monster	Puzzle: Infinite Corridor		Puzzle: Mayan Tablets		Puzzle: Room of Lights	
	Step 9: Involve the player	Closing argument: A good outcome is always possible		Closing argument: Having the freedom to decide is important	Closing argument: No matter how adverse the future looks, there will always be good things ahead		Closing argument: Things do not have to be perfect to be good		Closing argument: You have to avoid getting carried away	

### Evaluation

To explore the effects produced by our game, we compared *Bernstein* with an alternative protocol in a controlled intervention. For this purpose, we made use of a specialized instrument to measure assertiveness. In addition to that, to learn about the participants’ experience, a brief survey was conducted. With the information that we obtained, a quantitative analysis was conducted. To report our results, we followed the guidelines presented in the study by Montgomery et al [65].

### Ethics Approval

The study received prior approval from the Universidad de Colima ethics committee. All participants provided informed consent, confirming that they had read and understood the study’s purpose, procedures, potential risks, and their right to withdraw at any time without consequences. Participant data were collected and processed anonymously to ensure privacy. Students were informed that participation was voluntary, with no financial or material compensation provided.

### Evaluation Instrument

#### Overview

To assess participants’ abilities before and after the trial, we used the Spanish edition of the Rathus Assertiveness Schedule (RAS) [66,67]. This version was selected due to its successful implementation and validation throughout Latin America over the years. Furthermore, independent studies have consistently confirmed the RAS’s test-retest reliability, internal consistency, and validity [68,69]. The RAS comprises 30 items designed to evaluate a person’s assertiveness. Table 2 shows the classification of results based on the scores obtained.

**Table 2:** Classification of results.

Category	Score
Unassertive	–90 to –20
Situationally unassertive	–20 to 0
Mildly assertive	0 to 20
Assertive	20 to 40
Likely aggressive	40 to 90

#### Alternative Protocol

The alternative protocol we chose for the comparison was an adaptation of Your Perfect Right (YPR) [58]. YPR aims to contribute to the development of assertiveness-related skills by presenting and evaluating different scenarios (eg, dealing with difficult people, handling criticism, and expressing feelings) and by providing recommendations of best courses of action. We decided to compare our approach with YPR as it is regarded as the leading assertiveness training program and has been tested by independent studies throughout the years [70,71]. It should be noted that to save resources (eg, paper, printer ink, etc) and to facilitate the execution of the intervention, an informational website was created.

#### Participant Selection and Group Allocation

From January to December of 2022, students from 2 local high schools were invited to participate in our intervention. The selection criteria we established were (1) to be open to playing a serious game (previous experience with games not necessary) and (2) to have access to a computer with internet access. In total, 100 students accepted our invitation (65 boys and 35 girls). They were randomly and evenly divided into treatment and control groups through a blocked randomization process with block sizes of 4 people. The randomization was centrally managed to ensure an unbiased allocation. The average age of the participants was 17.59 years. In the end, only 92 participants finished the experiment. The main reason why most did not complete the study was a lack of time (eg, conflict with their class schedule). To thank students for their participation, they were offered extra credits in their computer science course (or its equivalent).

#### Development of the Intervention

The intervention consisted of a single 90-minute session, with both the treatment (*Bernstein*) and control (YPR) groups following identical timelines. Due to COVID-19 health concerns, all activities were conducted remotely.

At the start of the session, participants completed the Spanish edition of the RAS as a pretest to assess their interpersonal skills related to assertiveness before group allocation (treatment or control). Participants received a secure link with detailed instructions for accessing and completing the RAS. To ensure accurate responses, they were advised to find a quiet space, and additional measures were taken to enhance data reliability, including monitoring submissions for completeness and allowing only 1 submission per participant.

Following the pretest, participants engaged with either *Bernstein* (treatment) or a YPR session (control) for approximately 60 minutes. The session concluded with the posttest RAS assessment to measure any changes in assertiveness levels. To maintain the quality of the experiment, steps were taken to mitigate potential biases: (1) evaluator bias was minimized through automated data collection, and (2) demand characteristics were controlled by clearly explaining the intervention’s purpose to participants [72].

It is important to note that, throughout the study, no adverse events or unintended effects were observed.

## Results

### Treatment Group

A total of 46 students completed the intervention in the treatment group. To protect their personal information, we assigned each an identification code, which we used consistently throughout the study (T1-T46). The scores they obtained before and after playing can be seen in Table 3. To observe the effects produced by *Bernstein*, we compared the pre- and posttest scores of the participants using an inferential statistical test. The instrument we chose for this purpose, based on experts recommendations, was Student 1-tailed *t* test for paired samples [73,74]. Also known as the paired 1-tailed *t* test, this instrument was used to compare participants’ pre- and postintervention scores and assess the statistical significance of the observed differences. We established the following null hypothesis: there is no difference between participants’ scores before and after engaging with *Bernstein*. As shown in Table 3, the analysis yielded a *P* value below .05, suggesting that the intervention likely influenced the outcome.

**Table 3:** Pre- and posttest scores of participants in the treatment and control groups.

Group	Pretest, mean (SD)	Posttest, mean (SD)	*P* value
Treatment	–2.78 (25.93)	1.69 (29.48)	.01
Control	2.07 (25.69)	–2.39 (32.98)	.04

### Control Group

A total of 46 students completed the intervention in the control group. To protect their personal information, we assigned each one an identification code, which we used throughout the study (C1-C46). The scores they obtained before and after playing are shown in Table 3. To observe the effects produced by the alternative protocol, we compared the pre- and posttest scores of the participants using an inferential statistical test. The instrument we chose for this purpose was also Student 1-tailed *t* test for paired samples. The null hypothesis that we established was the following: there is no difference between the results of the participants before and after participating in a YPR session. As shown in Table 3, at *P*<.05, we obtained a statistically significant result.

### Statistical Evaluation of the Results

Following expert recommendations, we conducted a one-way ANOVA [73,74]. This analysis allowed us to compare the effectiveness of *Bernstein* and the alternative protocol by examining differences in score improvements between the 2 groups. The data that we used as input for this calculation were the average of the effects observed in both groups (ie, difference between pre- and posttest scores). The null hypothesis we established was the following: there is no difference between the results produced by *Bernstein* and the alternative protocol (ie, YPR) in the development of interpersonal skills related to assertiveness.

Table 4 shows the results of our calculations. The columns included in this table are sum of squares, degrees of freedom, mean square, *F* ratio, and *P* value of the null hypothesis being true. As Table 4 shows, at *P* value of <.05, there is a statistically significant difference between the 2 interventions, with the results favoring *Bernstein*.

**Table 4:** Comparison of the results of the treatment and control groups with a one-way ANOVA.

Results	SS^a^	*df*	MS^b^	*F^c^*	*P* value
Between groups	1809.39	1	1809.39	7.865	.006
Within groups	20702.56	90	230.02	—^d^	—
Total	22511.95	91	—	—	—

^a^SS: sum of squares.

^b^MS: mean square.

^c^*F*: *F* ratio.

^d^Not applicable.

### Participant Experience

To learn more about the impression that participants had of *Bernstein*, we asked them to respond to a form with both open-ended and 5-item Likert-scale questions (with 1 being totally negative and 5 totally positive). As the results suggest (Table 5), in general, participants enjoyed the intervention and had a pleasant experience. The highest rated factors were the language used in the game and the scenery design. The factor with the lowest score was the usability. When asked what suggestions they had to improve *Bernstein*, students mentioned the following: adding a checklist of the activities the character must do, including directional arrows to guide the character, adding more engaging sound effects, including side quests to improve the player’s game stats, and adding a navigation map.

**Table 5:** Evaluation of Bernstein.

Criterion	Value, mean (SD)
Usability	3.63 (1.21)
Language used	4.43 (0.91)
Narrative	4.09 (1.07)
Relationship with expectations	3.74 (1.11)
Character design	3.98 (1.06)
Scenery design	4.17 (1.07)
Navigation	3.74 (1.09)
Activities	3.72 (1.29)
Satisfaction	3.85 (1.06)
Experience	4.02 (0.89)

## Discussion

### Principal Findings

Bullying is a serious psychosocial problem that affects a significant number of young people. To help mitigate its consequences, we created *Bernstein*, a serious game that promotes the protective factor of assertiveness. To assess its effects, we carried out an exploratory trial with students from local high schools. As the results show, with *P*<.05, we obtained statistically significant results, confirming the feasibility of promoting assertiveness through *Bernstein*.

In contrast, despite having obtained a statistically significant value, the effects of a single YPR session (the alternative protocol) were moderately negative. YPR was originally designed to be delivered over multiple sessions, so compressing it into a single session likely contributed to its limited impact. Future research should explore alternative delivery protocols, including cumulative sessions, to better enhance assertiveness outcomes.

One way to estimate the effect size of an intervention, including those that seek to promote the protective factor of assertiveness, is to determine Cohen *d* for the values measured pre- and posttest [28,29,75]. Results of *d* can be interpreted as follows: 0.5 as small, from 0.5 to 0.8 as medium, and those 0.8 as large. For *Bernstein*, we obtained a Cohen *d* of 0.15885. These findings are consistent with results from similar interventions [28,29]. Although the effect size may be classified as small, research suggests that even modest improvements in assertiveness can yield meaningful real-world outcomes. Assertiveness enables students to resist peer pressure, navigate interpersonal conflicts, and express personal boundaries effectively, without resorting to aggression [29,57]. Consequently, incremental yet sustained gains in assertiveness have the potential to enhance resilience, strengthen peer relationships, and reduce the risk of bullying incidents.

To assess participant experience, we used a brief Likert-5 questionnaire. The highest rated features were language (4.43) and scenery design (4.17), while usability (3.63) and activities (3.72) scored lower, pointing to areas for improvement. Participants suggested adding activity checklists, directional arrows for navigation, and more engaging sound effects. These insights highlight key opportunities for future iterations. Improving usability will enhance player experience and better align game mechanics with mental health goals. Planned updates include clearer visual cues such as in-game maps, side quests to foster autonomy, new branching storylines to provide more opportunities for practicing assertiveness in varied contexts, and redesigned activities to sustain engagement and motivation.

While our study demonstrated the feasibility of promoting assertiveness through *Bernstein*, conducting the trial remotely introduced limitations. Without in-person guidance, participants missed opportunities for immediate feedback, which could have enhanced engagement. Real-time facilitator support, available in face-to-face settings, would allow for clearer guidance, encouragement, and correction during gameplay.

Although remote execution ensures safety and accessibility, follow-up studies should aim to compare remote and in-person interventions to better understand how delivery mode influences outcomes. A hybrid approach may ultimately strengthen *Bernstein*’s impact and scalability.

Another limitation of this exploratory trial is the relatively small sample size of the intervention, which restricts the generalizability of the findings. Future interventions with larger, more diverse participant groups are necessary to strengthen external validity and provide more conclusive evidence of *Bernstein*’s effectiveness.

*Bernstein* simulates realistic social situations, allowing players to practice expressing needs, setting boundaries, and managing peer pressure—skills directly relevant to school and social environments. Future research should explore the real-world impact of these skills through longitudinal studies, follow-up assessments, or hybrid interventions that combine gameplay with guided reflection or role-playing exercises.

While the RAS is a reliable tool for measuring assertiveness, its scope is primarily limited to behavioral expressions (ie, without explicitly evaluating underlying thought patterns or decision-making processes). Incorporating complementary metrics (eg, resilience, empathy, and cognitive or emotional regulation scales) would provide a more holistic assessment of *Bernstein*’s impact. Future studies should explore these additional dimensions to gain deeper insights into how the game influences players.

*Bernstein* is not the first serious game that seeks to contribute to the solution of the problem of bullying. Although there are other examples, to our knowledge, it is the only title that focuses on promoting the protective factor of assertiveness for that purpose. Future experiments with larger samples will be crucial to compare *Bernstein*’s effectiveness with other interventions and to validate its distinctiveness.

### Lessons Learned

The results obtained through the application of MCS were satisfactory. In its current iteration, it offers a comprehensive framework that facilitates the design of games capable of promoting protective factors as well as reducing risk factors, all while maintaining a targeted focus.

While serious games offer many benefits, they often fall short in replicating the complexities of real-world scenarios [76]. MCS, however, empowers creators to design games that more effectively capture and simulate nuanced, multilayered situations. As demonstrated in this study, it has proven to be an effective tool for crafting settings that reflect real-life challenges, enabling a more immersive and impactful experience for players.

We also identified areas for improvement. The Love Coin system functions as a cognitive-behavioral feedback mechanism, rewarding actions aligned with the game’s therapeutic goals. However, it may unintentionally bias player choices by encouraging reward-seeking over authentic exploration, leading players to prioritize earning coins over experimenting with diverse responses. This can limit deeper learning and reduce the sense of freedom, affecting the realism of their recovery journey. Future versions could implement more balanced scoring, rewarding effort, exploration, and varied strategies to promote meaningful engagement.

Furthermore, we assessed the benefits of implementing MCS for a specific issue. To ascertain its effectiveness to address different mental problems, additional validation would be required.

### Conclusions

Bullying, characterized by physical, verbal, and psychological aggression, continues to pose significant challenges in educational settings worldwide. As official data suggest, Mexico holds the highest global incidence of school bullying. This highlights the urgency of finding innovative and effective solutions to mitigate its impact on students’ well-being.

In response, we developed *Bernstein*, a serious game aimed at promoting assertiveness—identified as a key protective factor against bullying. Through the MCS methodology, grounded in CBT principles, *Bernstein* provides an interactive and immersive environment for players to develop interpersonal skills. The results of our intervention suggest that the game is a feasible tool for fostering assertiveness, with statistically significant improvements observed at *P*<.05.

While these findings are promising, it is essential to explore *Bernstein*’s efficacy in comparison with other bullying prevention interventions. Existing approaches, such as assertiveness training workshops, institutional awareness campaigns, and teacher-led interventions, have shown varying degrees of success. However, *Bernstein* offers a unique, scalable, and engaging alternative through a serious game, which may appeal to adolescents and allows them to practice assertive behaviors in a safe, controlled environment.

The potential for broader impact lies in integrating *Bernstein* into school curricula. Unlike traditional interventions that rely heavily on lectures or role-playing exercises, *Bernstein* immerses students in realistic situations, enabling experiential learning at their own pace. Furthermore, the game could complement other antibullying strategies, such as peer mentoring or mental health support services, providing a well-rounded toolkit for educators.

Future research should focus on expanding *Bernstein*’s implementation across diverse educational settings to evaluate its long-term impact and scalability. Collaborating with schools to integrate the game into regular curricula would allow for a more comprehensive evaluation of its effectiveness. Furthermore, comparative, “in-the-wild” studies with other antibullying programs could yield insights into how serious games such as *Bernstein* stack up against conventional interventions in promoting assertiveness and reducing bullying incidents.

The findings presented here showcase the feasibility of using *Bernstein* to promote assertiveness. While further validation with larger samples is needed, the results offer promising evidence that game-based interventions can play a meaningful role in mitigating bullying. With proper support and integration into educational practices, *Bernstein* has the potential to become a valuable tool in the ongoing effort to combat bullying and foster healthier school environments in Mexico and worldwide.

## Abbreviations

CBT: cognitive behavioral therapy
MCS: multiple composite scenarios
NPG: Ninja Pizza Girl
RAS: Rathus Assertiveness Schedule
RPG: role-playing game
YPR: Your Perfect Right

## Data Availability

Anonymized experimental data and the version of *Bernstein* used in this work are available for research purposes upon request.

## References

[1] Zhang S, Yu L, Wakefield RL, Leidner DE (2016). Friend or foe cyberbullying in social network sites. ACM SIGMIS. SIGMIS Database.

[2] Cohen R, Lam DY, Agarwal N, Cormier M, Jagdev J, Jin T, Kukreti M, Liu J, Rahim K, Rawat R, Sun W, Wang D, Wexler M (2014). Using computer technology to address the problem of cyberbullying. SIGCAS Comput Soc.

[3] de la Serna JM (2013). Causas del acoso escolar o bullying. Causes of bullying. WebConsultas.

[4] Kwak H, Blackburn J, Han S (2015). Exploring cyberbullying and other toxic behavior in team competition online games. https://doi.org/10.1145/2702123.2702529.

[5] Sutherland C, Coventry L, Sillence E (2014). Using animated scenarios to explore severity of cyberbullying and reporting readiness. Proceedings of the 26th Australian Computer-Human Interaction Conference on Designing Futures: the Future of Design (OzCHI'14).

[6] Zych I, Ortega-Ruiz R, Del Rey R (2015). Systematic review of theoretical studies on bullying and cyberbullying: facts, knowledge, prevention, and intervention. Aggression Violent Behav.

[7] Ayala F (2017). Bullying en México supera promedio de la OCDE. Bullying in Mexico exceeds the OECD average. Publimetro.

[8] (2021). México primer lugar en bullying: OCDE. Mexico ranks first in bullying: OECD. El Sol de Hidalgo.

[9] (2021). Suicidio infantil en México: un problema del que no se habla lo suficiente. Child suicide in Mexico: a problem that is not talked about enough. Pie de Página.

[10] Zych I, Farrington DP, Llorent VJ, Ttofi MM (2017). Protecting Children Against Bullying and Its Consequences.

[11] Lepe-Salazar F (2015). Preventing anorexia nervosa through cognitive-behavioral feedback in an affective game. https://doi.org/10.1109/HealthCom.2015.7454529.

[12] (2021). Eight examples that explain all you need to know about serious games and game-based learning. Gamelearn.

[13] Lepe-Salazar F, Salgado-Torres S (2023). Multiple composite scenarios: a game-based methodology for the prevention of mental disorders. Entertainment Comput.

[14] Balayn A, Yang J, Szlavik Z, Bozzon A (2021). Automatic identification of harmful, aggressive, abusive, and offensive language on the web: a survey of technical biases informed by psychology literature. Trans Soc Comput.

[15] Gaffney H, Farrington DP, White H (2021). Anti-bullying programs—toolkit technical report. Youth Endowment Fund.

[16] Iivari N, Ventä-Olkkonen L, Sharma S, Molin-Juustila T, Kinnunen E (2021). CHI against bullying: taking stock of the past and envisioning the future. https://doi.org/10.1145/3411764.3445282.

[17] Kinnunen E (2022). Tackling bullying with technology—a literature review of existing bullying prevention solutions. Oulun Yliopisto.

[18] Lievense P, Vacaru V, Liber J, Bonnet M, Sterkenburg P (2019). "Stop bullying now!" investigating the effectiveness of a serious game for teachers in promoting autonomy-supporting strategies for disabled adults: a randomized controlled trial. Disabil Health J.

[19] Stavroulia KE, Ruiz-Harisiou A, Manouchou E, Georgiou K, Sella F, Lanitis A (2016). A 3D virtual environment for training teachers to identify bullying. https://doi.org/10.1109/MELCON.2016.7495417.

[20] Bellini R, Olivier P, Comber R (2018). "That Really Pushes My Buttons": designing bullying and harassment training for the workplace. Proceedings of the 2018 CHI Conference on Human Factors in Computing Systems (CHI'18).

[21] Alm C (2007). The role of shyness and self-focused attention for attribution of reactions in social situations to internal and external causes. Scand J Psychol.

[22] Matsunaga M (2009). Parents don’t (Always) Know their children have been bullied: child-parent discrepancy on bullying and family-level profile of communication standards. Hum Commun Res.

[23] Tsaousis I (2016). The relationship of self-esteem to bullying perpetration and peer victimization among schoolchildren and adolescents: a meta-analytic review. Aggression Violent Behav.

[24] Wei C, Zhang H, Ye L, Meng F (2020). A school bullying detecting algorithm based on motion recognition and speech emotion recognition. https://doi.org/10.1109/ICHCI51889.2020.00066.

[25] Smith PK (2016). Bullying: definition, types, causes, consequences and intervention. Soc Pers Psychol Compass.

[26] Farrington D, Baldry A (2010). Individual risk factors for school bullying. J Aggress Conflict Peace Res.

[27] (2022). Assertiveness training. Association for Behavioral and Cognitive Therapies (ABCT).

[28] Nakamura Y, Yoshinaga N, Tanoue H, Kato S, Nakamura S, Aoishi K, Shiraishi Y (2017). Development and evaluation of a modified brief assertiveness training for nurses in the workplace: a single-group feasibility study. BMC Nurs.

[29] Omura M, Maguire J, Levett-Jones T, Stone TE (2017). The effectiveness of assertiveness communication training programs for healthcare professionals and students: a systematic review. Int J Nurs Stud.

[30] Wu YC (2018). BREAKAWAY: Examining the Educational Potential of Using a Narrative-Based Digital Game for Bullying Prevention.

[31] Calvo-Morata A, Alonso-Fernández C, Freire M, Martínez-Ortiz I, Fernández-Manjón B (2020). Serious games to prevent and detect bullying and cyberbullying: a systematic serious games and literature review. Comput Educ.

[32] Calvo-Morata A, Alonso-Fernández C, Freire M, Martínez-Ortiz I, Fernández-Manjón B (2021). Creating awareness on bullying and cyberbullying among young people: validating the effectiveness and design of the serious game Conectado. Telematics Inform.

[33] María E (2018). Del moral pérez and lourdes villalustre martínez análisis de "serious games" anti-"bullying": recursos lúdicos para promover habilidades prosociales en escolares. Rev Complut Educ.

[34] Ferreira Pc, Veiga Simão AM, Paiva A, Martinho C, Prada R, Ferreira A, Santos F (2021). Exploring empathy in cyberbullying with serious games. Comput Educ.

[35] Hall L, Jones S, Paiva A, Aylett R (2009). Fear Not! providing children with strategies to cope with bullying. https://doi.org/10.1145/1551788.1551854.

[36] Koli-Vehovec S, Smojver-Ai S, Dori TM, Ronevi Zubkovi B (2020). Evaluation of serious game for changing students' behavior in bullying situation. J Comput Assist Learn.

[37] Kriglstein S, Hengstberger F, Fribert F, Stiehl K, Schrank B, Pfeiffer A, Wernbacher T, Wallner G (2020). Be a buddy not a bully—two educational games to help prevent bullying in schools. https://doi.org/10.1145/3383668.3419914.

[38] Mancilla-Caceres JF, Amir E, Espelage D (2013). Adaptive game for reducing aggressive behavior. Proceedings of the companion publication of the 2013 international conference on Intelligent user interfaces companion (IUI '13 Companion).

[39] Raminhos C, Cláudio AP, Carmo MB, Carvalhosa S, Candeias MJ, Gaspar A (2015). A serious game-based solution to prevent bullying. https://doi.org/10.1145/2837126.2837135.

[40] Shome A, Rahman MM, Chellappan S, Al Islam ABMA (2020). A generalized mechanism beyond NLP for real-time detection of cyber abuse through facial expression analytics. https://doi.org/10.1145/3360774.3360793.

[41] Yoon HS (2020). A case study on the development of a serious game "Happy Class" for preventing cyber bullying. J Korea Game Soc.

[42] Lepe-Salazar F (2015). A model to analyze and design educational games with pedagogical foundations. https://doi.org/10.1145/2832932.2832951.

[43] Lepe-Salazar F (2021). Más allá de la diversiónxplorando el potencial de los videojuegos. Ludolab.

[44] Lepe-Salazar F, Cortés-Álvarez T, Serratos E, Jauregui-Flores L, Juárez-Cervantes E, Valdovinos-López R, Rincón-Martínez D, Moreno-De la Madrid R (2019). A game-based service to mitigate the risk of inundations caused by solid waste accumulation, Chapter 13, 22. Making Smart Cities More Playable.

[45] Disparity Games (2017). Ninja Pizza Girl.

[46] Stuart K (2014). Ninja Pizza Girl—a game about daughters and bullying. The Guardian.

[47] Gómez B (2016). A day at my school—denouncing bullying through games. On Serious Games.

[48] Pérez-Domínguez E (2015). Bully you, Bully Me – strategy party game about bullying and school shootings (2015). Civic Playground.

[49] Rockstar Vancouver (2006). Bully.

[50] Benites Paradeda R, Martinho C, Paiva A (2020). Persuasion strategies using a social robot in an interactive storytelling scenario. https://doi.org/10.1145/3406499.3415084.

[51] Charlton N, Kingston J, Petridis M, Fletcher B (2017). Using data mining to refine digital behaviour change interventions. https://doi.org/10.1145/3079452.3079468.

[52] Cheong YC, Khaled R, Grappiolo C, Campos J, Martinho C, Ingram GPD, Paiva A, Yannakakis G (2011). A computational approach towards conflict resolution for serious games. https://doi.org/10.1145/2159365.2159368.

[53] Eddy A (2019). Is technology killing human emotion? how computer-mediated communication compares to face-to-face interactions. https://doi.org/10.1145/3340764.3344451.

[54] Lappalainen PH (2020). Educating for diversity management in engineering. https://doi.org/10.1109/EDUCON45650.2020.9125329.

[55] Miller Frank N, Peters L (1998). Building trust: the importance of both task and social precursors. https://doi.org/10.1109/IEMC.1998.727781.

[56] Paradeda R, Ferreira M, Oliveira R, Martinho C, Paiva A (2019). The role of assertiveness in a storytelling game with persuasive robotic non-player characters. https://doi.org/10.1145/3311350.3347162.

[57] Reimold P (1996). Becoming more assertive in your communications. https://doi.org/10.1109/IPCC.1996.552589.

[58] Alberti R, Emmons M (2017). Your Perfect Right: Assertiveness and Equality in Your Life and Relationships.

[59] Hendrix K, van Herk R, Verhaegh J, Markopoulos P (2009). Increasing children's social competence through games, an exploratory study. https://doi.org/10.1145/1551788.1551823.

[60] Eng B (2017). Game-based learning to teach assertive communication ClickTalk for enhancing team play.

[61] van der Lubbe M, Gerritsen C, Formolo D, Otte M, Bosse T, Gentile C, Allegra M, Söbke H, Tibor Bosse H (2019). A serious game for training verbal resilience to doorstep scams. Games and Learning Alliance. GALA 2018. Lecture Notes in Computer Science.

[62] van der Lubbe L, Gerritsen C, Klein M, Hindriks K (2021). Empowering vulnerable target groups with serious games and gamification. Entertainment Comput.

[63] Beck JS (2011). Cognitive Behavior Therapy: Basics and Beyond (2nd edition).

[64] Leahy RL (2017). Cognitive Therapy Techniques: A Practitioner's Guide.

[65] Montgomery P, Grant S, Mayo-Wilson E, Macdonald G, Michie S, Hopewell S, Moher D (2018). Reporting randomised trials of social and psychological interventions: the CONSORT-SPI 2018 Extension. Trials.

[66] León Madrigal M (2009). Revisión de la escala de asertividad de rathus adaptada por León y Vargas (2009). Review of the Rathus Assertiveness Scale adapted by Leon and Vargas (2009). Rev Reflexiones.

[67] Rathus SA (1973). A 30-item schedule for assessing assertive behavior. Behav Ther.

[68] Gustafson R (1992). A Swedish psychometric test of the Rathus Assertiveness Schedule. Psychol Rep.

[69] Mann RJ, Flowers JV (1978). An investigation of the validity and reliability of the Rathus Assertion Schedule. Psychol Rep.

[70] Ingram JA, Salzberg HC (1990). Effects of in vivo behavioral rehearsal on the learning of assertive behaviors with a substance abusing population. Addict Behav.

[71] Omura M, Levett-Jones T, Stone TE (2019). Evaluating the impact of an assertiveness communication training programme for Japanese nursing students: a quasi-experimental study. Nurs Open.

[72] Dell N, Vaidyanathan V, Medhi I, Cutrell E, Thies W (2012). Yours is better! participant response bias in HCI. https://doi.org/10.1145/2207676.2208589.

[73] Box GEP, Hunter JS, Hunter WG (2005). Statistics for Experimenters: Design, Innovation and discovery.

[74] Lazar H, Feng JH, Hochheiser H (2017). Research Methods in Human-Computer Interaction.

[75] Cohen J (1988). Statistical Power Analysis for the Behavioral Sciences.

[76] Sparks M (2019). Metafocus: How Realistic Should Your Serious Game Be?. Learning guild.

